# The role of organizational citizenship behavior and patriotism in sustaining public health employees’ performance

**DOI:** 10.3389/fpsyg.2022.997643

**Published:** 2023-01-04

**Authors:** Mohammad Eqbal Rizaie, Emmanuel Mensah Horsey, Zhangzhi Ge, Nisar Ahmad

**Affiliations:** ^1^School of Public Affairs, University of Science and Technology of China, Hefei, Anhui, China; ^2^School of Management, University of Science and Technology of China, Hefei, Anhui, China

**Keywords:** organizational citizenship behavior, patriotism, employee performance, public health, sustainability

## Abstract

Employee performance during health crises is currently one of the most alarming global concerns. Owing to its significance, scholars assessed factors that improve such performance. However, such improvements in performance require public health employees’ discretionary behavior. We investigate the impact of organizational citizenship behavior and patriotism on employee performance, shifting the focus of the healthcare industry’s employee performance conversation to how it can be sustained. We used cross-section data from 541 Chinese public health employees who made up the final sample in this study, which has been shown to be appropriate for investigations utilizing a quantitative method. The results of our empirical analysis demonstrate that organizational citizenship behavior positively contributes to sustaining employee performance. We found the same to be true about the positive mediating effect of patriotism on the aforementioned relationship. The findings offer insights into how a country’s performance at both local and international levels propels and sustains the job performance of its citizenry. Thus, governments should be more efficient and ensure they maintain a robust institutional environment. This study provides theoretical and empirical evidence based on a research survey of the roles of organizational citizenship behavior and patriotism that sustain employee performance in China’s public health sector, in particular during health crises.

## Introduction

The increasing complexity of extreme events and their adverse effects on society has shifted the attention of scholars, governments, and organizations to strategically plan and counter future occurrences (Willis et al., 2021). The public health sector has received enormous pressure during extreme events such as the Ebola, SARS, and COVID-19 crises. These crises have necessitated the unwavering devotion of health employees (Willis et al., 2021). Amidst several factors, employee performance accentuates factors that relate to the role of health employees in overcoming the dilemma of disease outbreaks. Depending on the context, employee performance refers to the task or role performance relating exclusively to the completion of a task specified in a job incumbent’s task or job description (Pawar, 2013). In the literature, employee performance is one of the most important concerns for any organization (Motyka, 2018; Atatsi et al., 2019), and has been viewed as fundamental or in-role responsibility where employees are hired to perform in exchange for their compensation packages (Motyka, 2018; Atatsi et al., 2019). The importance accorded to employee performance has driven organizations’ continuous design of activities and study of behaviors in employees that could be tapped to improve their performance (Stoffers et al., 2014).

Employee performance has been studied in various contexts, across diverse disciplines and cultures in the past decades, with the aim of understanding behaviors, concepts, and resources that promote performance (Atatsi et al., 2019). However, a review of the literature showed factors that improve performance rather than those that sustain it. Precisely, prior studies emphasized factors that enable organizations to take advantage of, exploit and invest in employees’ physical, cognitive, and emotional capabilities. In this regard, research is required to provide insight into how employee performance can be sustained while taking into account prior emphases on issues such as employee burnout and role stress that develops when organizations focus on improving staff performance (Karatepe and Uludag, 2008; Prentice et al., 2013; Asgari et al., 2018; Virga et al., 2019; Lemonaki et al., 2021). Furthermore, due to the rise in lethal infectious diseases in recent decades and the critical role of public health personnel’s performance, this research focus requires further inquiry.

Driven by this importance, we examine the effects of organizational citizenship behavior and patriotism on employee performance in the Chinese public health sector. Extant studies looked into a variety of factors that influence employee performance. Sampling some recent studies reveal the works of van der Kolk et al. (2019) and Napitupulu et al. (2017). van der Kolk et al. (2019) investigated the effects of various types of management controls on public-sector employee performance. The study used motivation crowding and self-determination theories to claim that personnel, culture, action, and results control affect employees’ intrinsic and extrinsic motivation. These factors, according to the study, indirectly contribute to improved employee performance. Similarly, Napitupulu et al. (2017) investigated the impact of career advancement on public-sector employee performance. To strengthen this relationship, the authors investigated the mediating effects of perceived organizational support, job motivation, and affective commitment. They found that career development positively correlated with perceived organizational support, motivation, and affective commitment. Other assessments on enhancing employee performance revealed findings on employee burnout and role stress (Karatepe and Uludag, 2008; Prentice et al., 2013; Asgari et al., 2018; Virga et al., 2019; Lemonaki et al., 2021). To address this issue, the purpose of this study is to answer the following research questions: (1) How does health employees’ organizational citizenship behavior sustain public health employees’ performance during public health crises? (2) How does patriotism contribute to sustaining public health employees’ performance during public health crises?

The present study’s conceptualization shows that sustaining employee performance is essential to countering future epidemics that impose pressure on health service delivery. As a result, this study seeks to show that organizational citizenship behavior (which emphasizes extra-role and altruistic behaviors) and patriotism (which denotes an employee’s love and devotion for his/her country) are factors that sustain public health employees’ performance. Specifically, this study seeks to show the effect of health employees’ sacrificial and extra-role behavior on sustaining performance during a public health crisis. Drawing on extreme events such as COVID-19, the present study emphasizes the value of patriotism as a facilitator of employees’ willingness to perform tasks as a form of gratification for their country and the organization’s superiority. The unflinching support of public health employees is influenced mainly by altruistic behaviors. Cumulatively, this behavior can contribute to the quality of healthcare delivery to victims of these extreme events. Therefore, a country’s and public organization’s management acknowledgment of the essential role of these factors would facilitate improvements at the organization and country level which indirectly impacts employees’ extra-role and altruistic behaviors thereby sustaining employee health service delivery or performance during public health crises.

Organ (1988) defined organizational citizenship behavior as citizenship behaviors (altruism, conscientiousness, civic virtue, courtesy, and sportsmanship) that are not directly or openly acknowledged by formal incentives and that, when aggregated, support the company’s success. Researchers have recognized organizational citizenship behavior as an essential factor of performance, and it has sustained traction in the organizational literature (Chen et al., 2018). As a result, we expect the behaviors, for instance, altruism and conscientiousness that lie at the discretion of employees to sustain their health crises tasks performance. On the other hand, patriotism is a term that denotes a person’s emotional attachment to his or her country (Rupar et al., 2021). Prior studies emphasized that the core of patriotism is revealed in national and community leaders’ efforts, as well as mass media information dissemination, to assist citizens in their patriotic duty to combat the health crises (Rupar et al., 2021). Thus, we expect patriotic personnel to provide unflinching service delivery during healthcare emergencies. Put together, organizational citizenship behavior and patriotism should act as triggers of employee commitment which contributes to sustained performance.

This study makes two contributions to the existing body of knowledge. Theoretically, we contribute to the literature on organizational citizenship behavior by revealing the positive sustaining impact of organizational citizenship behavior on employee performance. Precisely, we show that health employees’ sacrificial and extra-role behaviors sustain performance during public health crises. In line with our assumption on the mediating role of patriotism, we enrich the literature by showing the conduit *via* which the link between organizational citizenship behavior and employee performance can be reinforced. Specifically, patriotism triggers employees’ exchange behavior. Put together, this supports the social exchange theory’s assertion that employees develop broad perspectives based on something given and reciprocate in a similar manner (Gouldner, 1960; Blau, 1964). In terms of empirical contribution, the study of Chinese public health employees’ organizational citizenship behavior and patriotism joins the growing body of empirical research in China on health crises and the role of government and organizations. However, we provide fresh insight into the roles of organizational citizenship behavior and patriotism in sustaining employee performance rather than prior studies’ central focus on the factors that improve it.

The subsequent sections of this study are organized as follows: Section 2 presents the theory and hypotheses. Section 3 reports the adopted methodology and how data were gathered. Section 4 presents data analysis. The final section presents the theoretical and empirical implications, limitations, and future research avenues and conclusions.

## Theory and hypotheses

### Social exchange theory

Social exchange theory provides a solid theoretical basis to understand how health employees’ performance is sustained. This is based on the importance employees accord to their organization. Economic exchange and social exchange relationships are two forms of exchange relationships that exist under the umbrella of social exchange theory (Blau, 1964). Economic exchange relationships greatly emphasize financial and tangible rewards that define what is exchanged and when reciprocation occurs (Blau, 1964; Cropanzano and Mitchell, 2005). In contrast, social exchange relationships pertain to the exchange of socio-emotional resources that are long-term orientated and arise from the importance employees attach to the needs of the other party (Blau, 1964; Cropanzano and Mitchell, 2005). The basic tenet of social exchange is the reciprocity rule, in which something offered by one party triggers an obligation of the other to return something in a similar way (Gouldner, 1960; Blau, 1964). Social exchange theory also describes the interchange of tangible/intangible and material/nonmaterial products between individuals (Lambe et al., 2001) which are embedded in the relationship between an organization and its employees (Cook et al., 2013).

In the public sphere, four core concepts of social exchange theory are articulated: (1) social outcomes that are produced by exchange interactions; (2) outcomes that are compared to alternatives over time to determine the exchange relationship’s dependence; (3) positive outcomes that increase trust and commitment over time; and (4) positive exchange interactions that produce relational exchange norms over time and governs the relationship (Martín Martín et al., 2018). In line with these core concepts, specifically, we propose that health employees having organizational citizenship behavior and patriotism engender feelings of obligation and trust which increases their commitment to the reciprocal rule (Gouldner, 1960; Blau, 1964). This is seen as unrelenting performance during health crises. Dominantly, social exchange theory has helped scholars establish insight into how employee exchange relationships resonate with the link between employee-level factors such as organizational citizenship behavior, leader-member exchange, employee trustworthiness, employee brand love, and employee performance (Cropanzano et al., 2017; Liaquat and Mehmood, 2017; Wang et al., 2019; Zhang et al., 2019; Wu et al., 2021). However, we lack insight into how social exchange theory unfolds in sustaining the relationships between organizational citizenship behavior, patriotism, and employee performance.

Through the lens of employee social exchanges (Cropanzano et al., 2017), our first hypothesis infers that health employees’ organizational citizenship behavior are intangible resource interchange of health workers of an organization. Since the health service industry is our prime focus, the intangible exchanges would comprise a commitment to tasks and interaction between employees to accomplish tasks. As a consequence, severe health crises, for instance, the health sector’s fight against the COVID-19 disease outbreak can be won through the lens of reciprocal offerings. Although during health crises, health employees’ concern about their health at the workplace may impair their performance (López-Cabarcos et al., 2020; Mani and Mishra, 2020), their sense of belonging ensures an inerratic flow of exchanges for better performance. We propose that the exchanges capture the five dimensions of organizational citizenship behavior (Cropanzano et al., 2017). Accordingly, organizational citizenship behavior becomes an exchange revealed by, for instance, employees’ devotion or stewardship, helping others to handle/accomplish assigned duties as well as exceeding their task performance levels during health crises.

Furthermore, to explain the mediating role of patriotism, we align the fundamental tenet of social exchange theory which emphasizes the reciprocity rule, in which something given triggers an obligation to return something equivalently (Gouldner, 1960; Blau, 1964). The effect of social exchange theory posits that health employees` love for their country is a source of emotions, and attempts to characterize the distinct emotional effects of different exchange structures to incorporate emotions as a core feature of social exchange processes, where a social exchange is conceptualized as a joint activity. Exchange outcomes produce emotions that vary in form and intensity, and can be either positive or negative. The theory predicts that the greater the shared responsibility, the stronger the emotions people will attach to the social units of the exchange. In service organizations, emotions influence how employees perceive and feel about their shared activity, their relationships, and their common group associations. According to the affect theory of social exchange, emotions are directed at the group context and are not limited to the service agent. In other words, the emotions resulting from social exchanges affect social relations, and a successful service encounter or relationship with a service employee will impact positively the entire service organization. Thus, patriotism becomes an exchange arising from employees’ emotional attachment to their country’s superiority. This emotional attachment would trigger the health employees’ obligation to contribute something that positively affects their country’s image. An avenue to do so is through the public health organization the employees serve. This idea is consistent with the social exchange theory’s claim on employees’ acceptance of “personal obligations, appreciation, and trust.”(Blau, 1964).

Given these proposed inferences, this study seeks to extend research on how social exchange theory relates to sustaining public health employees’ performance. We do so by emphasizing the connection between employees’ exchange behaviors comprising organizational citizenship behavior and patriotism. Last, we seek to contribute to the theory by providing insight into how patriotism serves as an exchange mediation mechanism in the link between organizational citizenship behavior and employee performance which has not gained this theoretical validation.

### Organizational citizenship behavior and employee performance

The concept of “organizational citizenship behavior” refers to a person’s discretionary behavior that is not immediately or openly recognized by the formal incentive system but that, in the aggregate, aids the organization’s effective functioning (Organ, 1988). Gary (2012) described organizational citizenship behavior as an employee’s voluntary conduct to perform duties outside of his or her allocated responsibilities for the progress or profit of their organization. Typically, organizational citizenship behavior has been studied from five dimensions consisting of altruism, courtesy, conscientiousness, civic virtue, and sportsmanship (Organ, 1988). Altruism details the behavior of assisting others in completing tasks. Courtesy details behaviors based on job-related problems, such as supporting co-workers who work inefficiently. Sportsmanship denotes the behaviors such as accepting unfavorable and less-than-ideal conditions. Civic virtue describes responsible behavior to partake in corporate life activities. Conscientiousness emphasizes commitment to tasks and achieving results that exceed expectations.

We claim that organizational citizenship behavior explains public health personnel’s maintaining commitment amid health crises when all five dimensions are taken into account. This is expected to unfold as follows: (1) we anticipate that altruism describes how health employees collaborate with their co-workers in completing tasks since such tasks are vital to their organization’s successful control of a health crisis; (2) courtesy should capture sacrificial behaviors such as collaborating with inefficient co-workers to resolve task-related issues in the interest of ensuring their organization efficiently handles a health crisis; (3) sportsmanship would describe the willing of health employees to maintain their performance amid unfavorable conditions such as their organization’s call for extra duties and working hours; (4) we expert civic virtue to take the form of employees unrelenting behavior to join corporate health agendas and taking actions to contain health crises; and (5) conscientiousness should be seen as health employees extra-role behaviors, for instance, as arising from their commitment that exceeds that performance quotas of their organization. Collectively, these assumptions resonate with earlier findings regarding the vital role of organizational citizenship behavior. For instance, organizational citizenship behavior has been emphasized as an absolute necessity in organizations and a system of collaboration and employees’ willingness to contribute and work to a cooperative system. In another word, organizational citizenship behavior is defined as extra work-related behaviors that go beyond the standard responsibilities listed in job descriptions and are assessed through formal assessments. This shows that organizational citizenship behavior is crucial to an organization’s process of reciprocal exchange and has a direct impact on employee performance (Sadeghi et al., 2016).

In light of the previous endorsement, it is obvious that earlier research has concentrated on elements that lead to sustained performance rather than on characteristics that contribute to employee engagement. Interestingly, Napitupulu et al. (2017)‘s findings provide avenues for future investigations into how and what factors contribute to sustaining employee performance in the public sector. According to earlier research, stress has a detrimental effect on nursing performance, which, in turn, has a detrimental effect on patient care and eventually results in high death rates (Sveinsdottir et al., 2006). Employees are significantly more engaged in the organization and spend more time there when they have a significant chance of meeting their requirements (Foss et al., 2015).

Furthermore, disease outbreaks have become a reality in the administration of healthcare operations, as well as in modern human resources management, with direct repercussions on employee tasks and adaptive performance (Nemteanu and Dabija, 2021). Employee task performance in healthcare can be defined as a set of behaviors that are linked to the organization’s goals and that each employee is responsible to accomplish. The recent global disease outbreak has pushed countries’ health systems to the limit, with unknown consequences for health workers’ task performance (Nemteanu and Dabija, 2021). Prior studies found several outcomes of organizational citizenship behavior, such as improved corporate productivity, maximized job effectiveness, and positive interaction between employees through active voluntary attitudes and behaviors (Rose, 2016; Gjurasic and Loncaric, 2018; de Geus et al., 2020). Similarly, organizational citizenship behavior enables an organization’s core business’s outstanding performance and catalyzes performance enhancements (Podsakoff et al., 2014). Prior studies reported other outcomes such as increased employee engagement in several industries such as the banking industry, hospitality industry, education, and service industry (Lyu et al., 2016; Zhang et al., 2017; de Geus et al., 2020). Thus, organizational citizenship behavior has been mainly linked to employees and organizational performance (Rose, 2016; Jiang et al., 2017). Given the role of organizational citizenship behavior, we expect unrelenting task performance during health crises. As a result, we suggest that:

*Hypothesis 1*: Organizational citizenship behavior positively relates to sustaining public health employees’ performance during public health crises.

### The mediating effect of patriotism

This study posits that patriotism is an outstanding factor that propels health employees’ organizational citizenship behavior to sustain employee performance. This proposition follows patriotism’s emphasis on an individual’s love and commitment to his or her country (Sekerdej and Roccas, 2016; Marzecki, 2020). Therefore, our argument on the possibility of sustaining health employees’ performance is triggered by their love for their love which results in an unflinching commitment to their job roles during life-threatening events such as deadly epidemics. This study focuses on a more comprehensive multidimensional notion of employee patriotism. Indeed, research indicates that various forms of employee patriotism are associated with varying degrees of social participation (Rupar et al., 2021). Notably, patriotism, defined as an emotional attachment to one’s nation, has been found to be an excellent predictor of time and effort invested in tasks that benefit the country (Sekerdej and Roccas, 2016) and various civic engagement activities (Rupar et al., 2021). Having a sense of belonging and personal duty toward one’s home country and one’s fellow citizens are indicative of patriotism in individuals. Accordingly, those who have a strong sense of national identity may have a greater sense of personal duty to act, which could, in turn, influence attitudes and behavioral reactions, including those connected to the COVID-19 situation (Everett et al., 2020). Individuals who have a strong sense of belonging to their country may also have a strong sense that the state is responsible for defending its inhabitants during a crisis. Individual responses aimed at preventing future harm have been demonstrated in previous research (Iqbal and Bilali, 2018), which suggests that acknowledging the responsibility of one’s country is important. Consequently, not only does the sense of individual responsibility play a role in the issue, but also so does the sense of country or state accountability, which may help to explain the links between national identification, attitudes, and behaviors in the COVID-19 dilemma. In keeping with these findings, we anticipated that patriotism would be a strong or consistent predictor of attitudes and behaviors connected to a disease outbreak (e.g., COVID-19).

In the literature, patriotism is also emphasized as national identification which is an individual’s emotional inclination to his/her country (Rupar et al., 2021). Patriotism has been studied in different spheres for instance in military sacrifice, politics, populace tax compliance, history, and during extreme events specifically its current occurrence in COVID-19-related studies. In addition, the essence of patriotism is revealed in the effort of national and community leaders and mass media information dissemination to facilitate citizens’ patriotic duty to fight against the COVID-19 pandemic (Rupar et al., 2021).

**Figure 1:**
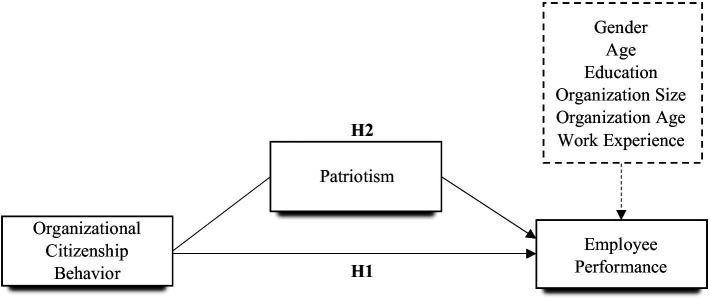
Research model.

The literature documented that patriotism comes in three forms comprising conventional, constructive, and glorification. Care, emotional attachment, and love toward one’s country and people describe an individual’s conventional patriotism (Sekerdej and Roccas, 2016). However, the literature posits that one’s emotional attachment and love toward his/her country alone cannot represent patriotism. This shed light on the significant emphasis placed on the constructive and glorification forms of patriotism. The serious reflection, motivation, and devotion that influence an individual to work to better his/her country explain constructive patriotism (Sekerdej and Roccas, 2016). Constructive patriotism motivates individuals to participate in a variety of political and social activities that benefit their country and fellow citizens (Rupar et al., 2021). Additionally, constructive patriotism is forward-looking and is more likely to promote long-term goals (Rupar et al., 2021). Glorification is defined as a belief in the nation and state based on political and geographical traits (Leidner et al., 2010), and results in the formation of extrinsic group boundaries (Schatz, 2020). Also, a recent study found that glorification is associated with increased support for quick, short-term interventions (Rupar et al., 2021). Furthermore, glorification is associated with employee national conservatism (Jost et al., 2003), which is characterized by a reduced willingness to participate in acts that could affect the country, such as protests or avoiding not cooperating during major disease outbreaks. Thus, we anticipated that glorification would be associated with decreased support for such efforts and, as a result, with decreased rates of compliance with hygienic and social norms. Glorification patriotism details the love which is accompanied by unquestioning, blind devotion to one’s nation’s structures and policies, and one’s thinking of the nation’s superiority. Therefore, this study’s argument regarding the mediating effect of patriotism is anchored on its three forms and its pivotal role in shaping employees’ organizational citizenship behavior and performance. This also follows prior studies’ emphasis on the effect of patriotism (national identification) in shaping behaviors and the need to consider studying the joint roles of the three forms of patriotism (Rupar et al., 2021; Figure 1). Based on these arguments, the proposed study hypothesizes that:

*Hypothesis 2*: Patriotism positively mediates the relationship between organizational citizenship behavior and employee performance such that it sustains the relationship.

## Materials and methods

### Sampling and data collection

The population sample of interest in this study was a cross-section of Chinese public health workers. The final sample was 541, which is proven suitable for studies using a quantitative approach (Hair et al., 2014). The sample consisting of health employees’ organizations (e.g., hospitals) was gathered from a Chinese public organizations database. We used a questionnaire to gather the data from this sample. To determine our study participants, experts inside and outside the University of Science and Technology of China were consulted. After receiving the affirmation of the hospitals on data collection through the University of Science and Technology of China, the questionnaire was disseminated to the hospitals where the health employees were combating the COVID-19 disease outbreak and all the participants ensured that data were kept confidential and anonymous. We conducted a back-translation procedure following Brislin (1970) to translate the English questionnaire into a simplified Chinese script. Proposed an iterative procedure of repeated independent translation and back-translation by a translator team. This method necessitates the use of numerous independent bilingual translators (Triandis and Brislin, 1984). A bilingual translator blindly translates an instrument from the original language to the target language; a second bilingual translator independently reverses the translation from the target language to the original language. The two versions of the instrument (original language and back-translated version) are then compared for concept equivalence. When an error in the back-translated version is discovered, another translator retranslates the item. This process is repeated until a team of bilingual translators determines that the two versions of the instruments are identical and include no grammatical problems. The translation team was made up of three purposefully selected professors from the authors’ university, including two Ph.D. students were had a mastery command of the English and Chinese Mandarin languages.

Furthermore, the Chinese version was tested on five Chinese public health officials, and the questionnaire was readjusted based on their feedback. Along with this procedure, our Chinese-translated questionnaire was equivalent to the English version. The final questionnaire consisted of four main parts: (1) sample demographic information; (2) questions related to employees’ organizational citizenship behavior; (3) questions related to employees’ patriotism; and (4) employee performance. To avoid ambiguity and response bias, each section of the questionnaire was preceded by instructions (Podsakoff et al., 2003). Respondents were assured that there were no right or wrong answers and that all responses would be treated with the utmost confidentiality. The survey lasted for 5 months from August 2021 to December 2021, during which questionnaires were administered online using e-mail and WeChat.

We engaged and used 541 valid responses to empirically test this study’s proposed relationships. To arrive at these valid responses, we included several “trap” questions, asking about their OCB experience during the COVID-19 crisis. In particular, we added the following: “*I have helped my other co-health workers who have been absent during the COVID-19 crisis*”; *and* “*During the past 8 months, I have voluntarily helped other co-health workers during the COVID-19 crisis*.” The logic behind asking these questions was to check whether a respondent was paying attention while answering the survey and the consistency of their answers. To ensure the suitability of our sample size, we conducted a sample adequacy test. This assessed how the factors linked to one another (the strength of the partial correlation between variables; Kaiser, 1974). As shown in Table 1, the Kaiser-Meyer-Olkin Measure (KMO) value of 0.896 met the threshold of >0.50, implying that the sample size is sufficient to test the study’s hypotheses. Furthermore, regarding our sample demographic statistics, 54.5% of the 541 valid responses were female and 45.5% male. Table 2 reports the sample statistics in detail.

**Table 1:** Sample adequacy test.

KMO and Bartlett’s test, *N* = 541		
Kaiser-Meyer-Olkin measure of sampling adequacy		**0.896**
Bartlett’s test of sphericity	Approx. Chi-square	29250.906
	*df*	990
	Sig.	0.000

KMO value is significant at >0.50. KMO value bolded.

**Table 2:** Sample properties – *N* = 541.

Variable	Category	Frequency	Percent
Gender	Male	246	45.5
	Female	295	54.5
Age	18–24	94	17.4
	25–34	209	38.6
	35–44	157	29.0
	45–54	62	11.5
	55–64	19	3.5
Education	Diploma	80	14.8
	Bachelor	327	60.4
	Masters	128	23.7
	Doctorate	6	1.1
Organization Age	0–10	331	61.2
	11–20	140	25.9
	21–30	47	8.7
	31–40	15	2.8
	41–50	7	1.3
	51+	1	0.2
Organization Size	Small (10–49)	389	71.9
	Medium (50–249)	127	23.5
	Large (above 250)	25	4.6
Working Experience	Less than 2 years	106	19.6
	3–5 years	150	27.7
	6–10 years	79	14.6
	Greater than 10 years	206	38.1

### Measurement scale

All constructs were measured following prior studies published in social sciences citation index (SSCI) journals.

#### Organizational citizenship behavior

Organizational citizenship behavior (OCB) was measured using 14 items (Angeles Lopez-Cabarcos et al., 2020). For instance, the items measuring organizational citizenship behavior are (1) I helped health employees who have heavy workloads; (2) I helped orient new health employees even though it is not required; and (3) I have helped my other co-health workers who have been absent during COVID-19 crisis.

#### Patriotism

Patriotism was measured following the works of Roccas et al. (2006) and Sekerdej and Roccas (2016). An aggregate of eight items measured patriotism. Patriotism is a high-level construct with three low-level dimensions comprising conventional, constructive, and glorification. The items were refined to fit the context of the current study and assessed by experts. Three items measured the conventional dimension of patriotism. Sample items are (1) I like China; (2) I love my country; and (3) the fact that I am Chinese is an important part of my identity. In addition, the constructive dimension of patriotism had three items: (1) I oppose some policies because I care about my country and want to improve it; (2) I express my attachment to China by supporting efforts aimed at positive change; and (3) People should work hard to move this country in a positive direction. Two items measured the glorification dimension of patriotism. Sample items are (1) my nation is better than other nations in all aspects and (2) it is disloyal to criticize China.

#### Employee performance

We adapted 23 items from the work of Pradhan and Jena (2016) to measure employee performance. The items were refined to fit the context of health employees and thoroughly assessed by experts. For instance, (1) I use to maintain a high standard of work during public health crises; (2) I use to perform well to mobilize collective intelligence for effective teamwork to overcome during public health crises; and (3) I used to extend help to my co-health workers when asked or needed. These three items indicate the three dimensions of employee performance: task performance, adaptive performance, and contextual performance, respectively. Each of these measurement items was rated on a 7-point Likert scale ranging from 1 = strongly disagree to 7 = strongly agree. As shown in Table 2, gender, age, education, work experience, organization age, and organization size were controlled in this study due to their influence on employee task performance (Pradhan and Jena, 2016).

### Data analysis and results

#### Exploratory factor analysis

Exploratory factor analysis (EFA) was used in this study to determine the reliability, convergent, and discriminant validity of constructs/items using SPSS software version 24. As shown in Table 3, the Cronbach’s alpha (CA) coefficient estimated the reliability of all constructs. The Cronbach’s alpha of organizational citizenship behavior, patriotism, and employee performance was 0.903, 0.874, and 0.951, respectively, which indicated that the reliability of the whole items was above the cutoff point of 0.70 for significance (Hair et al., 2017). In addition, all constructs’ average variance extracted (AVE) values were above the cutoff point of 0.5, indicating that these constructs had acceptable convergent validity (Hair et al., 2017). The final constructs’ validity test compared the AVE of each variable/construct with the square of their respective correlation coefficient. The AVE values were found to be higher than the square of the correlation coefficient, which inferred that discriminant validity met the recommended requirement (Fornell and Larcker, 1981). To improve the scale validity, constructs/items with loadings below the recommended threshold of 0.60 (Hair et al., 2010) were eliminated during the analyses.

**Table 3:** Constructs/items measurement properties.

Organizational citizenship behavior		CA	CR	AVE
OCB2	0.721	0.903	0.938	0.628
OCB3	0.826			
OCB4	0.798			
OCB5	0.848			
OCB7	0.749			
OCB9	0.723			
OCB10	0.691			
OCB12	0.879			
OCB13	0.873			
**Patriotism**		0.874	0.921	0.626
CPA1	0.765			
CPA2	0.719			
CPA3	0.687			
CPB1	0.861			
CPB2	0.814			
CPB3	0.852			
CPC1	0.823			
**Employee performance**		0.951	0.979	0.676
TP1	0.862			
TP2	0.753			
TP3	0.907			
TP4	0.832			
TP5	0.729			
TP6	0.819			
AP7	0.883			
AP8	0.869			
AP9	0.799			
AP10	0.800			
AP11	0.833			
AP12	0.822			
AP13	0.804			
CP14	0.786			
CP15	0.897			
CP16	0.814			
CP17	0.792			
CP18	0.746			
CP19	0.851			
CP20	0.745			
CP21	0.745			
CP22	0.848			
CP23	0.932			

#### Common method bias and multicollinearity analysis

Further robustness checks focused on assessing common method bias and multicollinearity issues. The study used Harman’s one-factor test to eliminate the risk of common technique bias (Podsakoff et al., 2003). A common technique bias emerges when a single factor accounts for more than 50% of the variance (Harman, 1967). The statistical results obtained were below the recommended cutoff point of 50% (Harman, 1967). Another way to assess common method bias is to use the variance inflation factor (VIF) to check for multicollinearity among constructs. The resultant highest VIF value of 4.682 was less than the threshold value of 10% (Neter et al., 1990). As a result, no common method bias and multicollinearity are not a problem in our study.

#### Descriptive statistics and bivariate correlation analysis

The strength of the linear association between organizational citizenship behavior, patriotism, and employee performance was determined using Pearson’s bivariate correlation analysis. Table 4 reports the statistically significant relationship among all variables.

**Table 4:** Descriptive statistics and correlations.

Variables	1	2	3	4	5	6	7	8	9
Gender	1								
Age	−0.183**	1							
Education	0.214**	−0.161**	1						
Organization Age	−0.266**	0.260**	0.040	1					
Organization Size	−0.136**	0.114**	0.125**	0.307**	1				
Working Experience	−0.172**	0.877**	−0.063	0.315**	0.094*	1			
OCB	−0.156**	−0.154**	−0.094*	0.094*	0.045	−0.123**	1		
P	−0.009	0.002	0.039	0.079	−0.056	0.058	0.317**	1	
EP	−0.051	−0.105*	−0.051	0.081	0.085*	−0.118**	0.449**	0.267**	1
Means	1.55	2.45	2.11	1.58	1.33	2.71	5.30	5.71	5.03
SD	0.498	1.018	0.646	0.882	0.560	1.167	1.098	0.978	0.937
VIF	1.179	4.682	1.157	1.293	1.154	4.659	1.218	1.149	

*N* = 541; **Correlation is significant at the 0.01 level (2-tailed). *Correlation is significant at the 0.05 level (2-tailed). OCB, organizational citizenship behavior; P, patriotism; EP, employee performance.

### Hypotheses testing

The two hypothesized relationships were tested through four hierarchical regression models, as shown in Table 5. In all four models of the analysis, employee performance was the dependent variable. All controlled variables were entered in all four regression models following prior studies’ endorsements of their possible influence on employee performance. Model 1 calculated the effect of the control variables on employee performance. Statistically significant values were obtained for the control variables: education, organization age, and organization size except for age, gender, and work experience. In Model 2, the regression coefficient (*β* = 0.436, *p* < 0.001) showed a positive relationship between organizational citizenship behavior and employee performance. This infers that this study’s *Hypothesis* 1 is valid. Model 3 reports the result (*β* = 0.158, *p* < 0.001) obtained for the effect mediating of patriotism on the relationship between organizational citizenship behavior and employee performance. The result demonstrated that *Hypothesis* 2 is true. Model 4 estimated the interaction effect: organizational citizenship behavior and patriotism (OCB x P). We had statistically significant interaction effects for this estimation. This further validated *Hypotheses* 2.

**Table 5:** Hierarchical linear regression.

	Employee performance (dependent variable)				
Variables	Model 1	Model 2	Model 3	Model 4	Interpretation
**Controls**	*β*	*β*	*β*	*β*	
Gender	−0.027	0.034	0.027	0.025	
Age	−0.052	0.065	0.079	0.078	
Education	−0.075*	−0.026	−0.037	−0.017	
Organization Age	0.105**	0.058	0.050	0.062	
Organization Size	0.076*	0.061	0.076*	0.076*	
Working Experience	−0.122	−0.141*	−0.170**	−0.155**	
**Main Effect**					
Organizational Citizenship Behavior		0.436***	0.383***	1.110***	H1 supported
**Mediator**					
Patriotism			0.158***	0.706***	H2 supported
**Interaction**					
OCB x P				1.046**	
*R* ^2^	0.040	0.215	0.237	0.250	
Adjusted *R*^2^	0.029	0.205	0.225	0.237	
F	3.700***	20.857***	20.613***	19.622***	

*N* = 541; *p < 0.1, **p < 0.05, ***p < 0.001. OCB, organizational citizenship behavior; P, patriotism. Hypotheses testing coefficients are bolded.

To further validate the mediation effect of patriotism, we employed the step-by-step instructions recommended by Zhao et al. (2010). This mediation approach is more appropriate since it corrects the constraints associated with previous mediation analytical procedures. Zhao et al. (2010) demonstrated that the significance of the indirect effect of a x b three-variable causal model is the only prerequisite for predicting mediation. This eliminates the necessity for an initial test of the significance of the X-Y variables. Other researchers, for instance, Rucker et al. (2011) stated that a significant link between X and Y is not required for assessing mediation. Zhao et al. (2010) also suggested that the bootstrapping technique is more appropriate to calculate the significance of a × b three-variable causal model. Along with this insight, this study estimated the strength of the mediation effect of patriotism on the link between organizational citizenship behavior and employee performance. Table 6 reports the acceptable results of the bootstrapping technique.

**Table 6:** Mediation analysis.

Path	Estimate	95% confidence interval		Result
		Lower level	Upper level	
OCB → P → EP	0.0440	0.0207	0.0706	Supported

OCB, organizational citizenship behavior; P, patriotism; EP, employee performance.

## Discussion

### Theoretical and empirical implications

The goal of this study was to examine the roles of organizational citizenship behavior and patriotism in sustaining public health employees’ performance during health crises. Throughout the COVID-19 disease outbreak, hospital staff encountered a range of challenges, including a significant work overload that impairs their capacity to perform in hospitals (Aguiar-Quintana et al., 2021). Health workers are a critical component and play a vital role in the delivery of healthcare to patients in both public and private hospitals. While China’s public hospitals are now more overcrowded than usual due to the COVID-19 outbreak, the chaos has deleterious effects on healthcare employees’ work performance, such that it places significant pressure on healthcare employees’ work performance. Prior investigations and discoveries on the factors that improve employee performance have been driven to an extent by this fundamental focus (Napitupulu et al., 2017; van der Kolk et al., 2019). Despite the importance of those contributions, some studies found that improving employee performance leads to employee role stress, burnout, and turnover (Karatepe and Uludag, 2008; Prentice et al., 2013; Asgari et al., 2018; Virga et al., 2019; Lemonaki et al., 2021). In light of this, we conducted a literature review and discovered fewer insights into factors that sustain employee performance. In this effect, we posed the research questions: (1) How does health employees’ organizational citizenship behavior sustain public health employees’ performance during public health crises? (2) How does patriotism contribute to sustaining public health employees’ performance during public health crises? These questions were addressed in light of recent rapid health crises and the increased focus on the health sector’s performance (Willis et al., 2021).

To address this gap, this study conceptualized that organizational citizenship behavior and patriotism are factors that can help employees sustain performance in the healthcare industry. To be more precise, this study focuses on the interaction of employees’ organizational/national level characteristics that automatically instill a desire in employees to continue providing excellent service despite their fear of negative repercussions, for instance, the perceived risk associated with COVID-19 (Yu et al., 2021). Emphasis is placed on active and voluntary attitudes and behaviors of employees to enhance organizational productivity (Kim et al., 2013). Organizational citizenship behavior enhances organizational productivity, increases job effectiveness, and maximizes positive interaction between employees through active and voluntary attitudes and behaviors of employees (Kataria et al., 2012). Overall, considering the characteristics of organizational citizenship behavior, it can be established that organizational citizenship behavior enables outstanding performance, thus, can serve as a sustaining mechanism of employee performance. Chiaburu et al. (2013) indicated that organizational citizenship behavior induces: helping, taking charge, and creative behavior. Specifically, helping means supporting the work of fellow employees and sharing necessary information, taking charge implies pursuing change and innovation, while creative behavior involves presenting creative ideas for the organization. Along with this insight, this study’s proposed relationships were tested to ascertain whether it echoes the prior assertions above.

Theoretically, the results validated this study’s hypothesis that organizational citizenship behavior sustains employee performance as well as the positive mediating effect of patriotism. Both organizational citizenship behavior and patriotism had the same strong positive effect on employee performance. These findings make contributions to the organizational citizenship behavior literature on the beneficial role of health employees’ citizenship behavior revealed as altruism, courtesy, conscientiousness, civic virtue, and sportsmanship behaviors (Organ, 1988; de Geus et al., 2020). In addition, this finding can explain public health employees’ unwavering service delivery during extreme events such as Ebola, Influenza, SARS 2003, and COVID-19 (Rupar et al., 2021).

Following the recommendation of prior studies on the need to investigate the combined influence of the three components of patriotism (Rupar et al., 2020, 2021), we add to the literature by identifying patriotism (conventional, constructive, and glorification) as a facilitator of public health employees’ willingness to maintain performance during health crises. Last, the favorable effects of organizational citizenship behavior and patriotism contribute to the literature on how employee performance can be sustained (maintained productivity) rather than improved (increased productivity; Atatsi et al., 2019). Overall, this insight lends credence to social exchange theory’s emphasis (four core principles) on the essence of employee social exchanges and its outcomes (Martín Martín et al., 2018). We elaborate on this study’s contribution to the social exchange theory in two ways. First, no study has provided insight into how the social exchange theory (Blau, 1964; Cropanzano and Mitchell, 2005) affects employee sustained performance. Although prior studies emphasized the factors responsible for employee performance (Cropanzano et al., 2017; Liaquat and Mehmood, 2017; Wang et al., 2019; Zhang et al., 2019; Wu et al., 2021), the literature lacks insight into how social exchange theory unfolds in sustaining the relationships between organizational citizenship behavior, patriotism, and employee performance. Thus, we extend the social exchange theory (Blau, 1964; Cropanzano and Mitchell, 2005) by identifying organizational citizenship behavior and patriotism as what employees offer in exchange for the value of their organization and country.

Second, the mediating effect of patriotism further provides fresh insight into the mechanism *via* which the sustaining effect of organizational citizenship behavior on employee performance is strengthened. This proposition follows patriotism’s emphasis on an individual’s love and commitment to his or her country (Sekerdej and Roccas, 2016; Marzecki, 2020). Accordingly, health employees who have a strong sense of national identity may have a greater sense of personal duty to act, which could, in turn, influence attitudes and behavioral reactions, during the COVID-19 situation (Everett et al., 2020). Although there is ample evidence of the positive relationship between organizational citizenship behavior and employee-related performances (Rose, 2016; Gjurasic and Loncaric, 2018; de Geus et al., 2020), no evidence exist about how it is sustained and the black box mechanisms that mediate this nexus.

Empirically, the utilization of Chinese health practitioners in this study provides empirical evidence to explain the performance of the Chinese health sector from the perspective of the positive contributory effect of organizational citizenship and patriotism, which received no research attention. Precisely, we show that when Chinese health employees engaged in organizational citizenship behavior and patriotism they stay committed to their tasks regardless of the unfavorable conditions they face during health crises. These behaviors: organizational citizenship behavior and patriotism are what they offer to sustain performance. These findings have practical implications for healthcare executives as well as national governments. First, managers of organizations should place a premium on health employees’ intrinsic job satisfaction to increase their organizational citizenship behavior. Rather than delivering extrinsic rewards, managers should provide intrinsic benefits (e.g., job meaningfulness, job responsibilities, and job challenge) and work to boost employees’ sense of intrinsic happiness. Finally, maintaining employees’ patriotism should be a top concern for the government *via* an effective institutional role in national growth. This should serve as a long-term support system for employees’ employment performance. As a result, robust policies, programs, and other crucial resource allocations should be the hallmark of government. Such prioritization should include a national health-crisis preparedness strategy to impact the performance of healthcare employees.

### Limitations and suggestions for future research

The limitations of this study include its cross-sectional design which prevents us from concluding the causal relationships among the studied variables and how the sustainability of employee performance can be measured over a time frame. Future research should employ other data types (time series/panel). Also, our sample was limited to the Chinese context which may limit the generalizability of the results. Due to similar adverse effects of deadly diseases and the essential role of health employees’ performance, comparative analyses could be conducted to understand the similarities and differences across countries using these data types. Collectively, this approach would help generalize results. In addition, scholars could examine additional variables to broaden understanding. Specifically, further research could be directed toward the exploration of potential moderators, such as those related to the organizational context, as well as other contextual factors such as cultural and institutional dimensions of China in the link between organizational citizenship behavior and employee performance. These could be addressed from two standpoints. First, this study’s arguments were centered on employees’ sustaining exchanges arising from their organizational citizenship behavior and patriotism. As a result, we begin a conversation about understanding factors that sustain employees’ performance rather than ones that enhance it. Thus, future research could assess how human resource and organizational behavioral factors, for instance, leadership inclusive behavior; good management and leadership; perceived organizational support; and a good working environment sustains employee performance. Second, the cultural dimensions of high collectivism, power distance, and high preference for avoiding uncertainty are different from those of individualist societies. Subsequent research should be carried out in contexts dissimilar to China to improve the generalization of our findings.

## Conclusion

This study assessed the effect of organizational citizenship behavior and patriotism on employee performance. The empirical analysis used 541 Chinese public health employees to validate the effect of these factors. The primary focus has influenced earlier research and discoveries on the elements that boost employee performance to some extent (Napitupulu et al., 2017; van der Kolk et al., 2019). Despite the value of those efforts, some research revealed that elevating employee performance causes job stress, burnout, and turnover (Park et al., 2020). As a result, we reviewed the literature and found fewer insights into the variables that support employee performance. This study developed the concept that organizational citizenship behavior and patriotism are elements that can support employees’ performance in the healthcare sector. To be more specific, this study focuses on the interaction of employees’ overall organizational characteristics that inevitably instill a desire in employees to continue offering outstanding service despite their fear of adverse consequences, for instance, the perceived risk associated with COVID-19 (Özlü et al., 2022). We found a strong positive impact of organizational citizenship behavior and patriotism on employee performance. In line with the social exchange theory, we established these connections. Consequences in practice shed light on peak performance in organizations and governments to ensure that the investigated factors continue to impact employee tasks performance amid national health crises. This study contributes to the related literature on factors that sustain employee performance during health crises rather than existing research’s focus on its improvement. Following the employment of a cross-sectional design, conceptual model, and measuring items in this study, recommendations for future research are reported.

## Data availability statement

The raw data supporting the conclusions of this article will be made available by the authors, without undue reservation.

## Ethics statement

Ethical review and approval was not required for the study on human participants in accordance with the local legislation and institutional requirements. The patients/participants provided their written informed consent to participate in this study.

## Author contributions

MR and EH contributed to the conception and design of the study. MR wrote the abstract, theory, and hypothesis sections. EH wrote the introduction, discussion, and conclusion sections. ZG and NA organized the data collection, conducted the statistical analysis, and handled the methodology and data analysis. MR, EH, ZG, and NA partook in proofreading and revision of the paper. All authors contributed to the article and approved the submitted version.

## Funding

This study was supported by the Fundamental Research Funds for the Central Universities “The Combination Model and Implementation Path of Medicine-Nutrition-Science-Education in the Big Health Environment” (WK2160000008).

## Conflict of interest

The authors declare that the research was conducted in the absence of any commercial or financial relationships that could be construed as a potential conflict of interest.

## Publisher’s note

All claims expressed in this article are solely those of the authors and do not necessarily represent those of their affiliated organizations, or those of the publisher, the editors and the reviewers. Any product that may be evaluated in this article, or claim that may be made by its manufacturer, is not guaranteed or endorsed by the publisher.
